# Activation of PPARs Modulates Signalling Pathways and Expression of Regulatory Genes in Osteoclasts Derived from Human CD14+ Monocytes

**DOI:** 10.3390/ijms20071798

**Published:** 2019-04-11

**Authors:** Abe Kasonga, Marlena C. Kruger, Magdalena Coetzee

**Affiliations:** 1 Department of Physiology, University of Pretoria, Pretoria 0001, South Africa; magdalena.coetzee@up.ac.za; 2 School of Health Sciences, College of Health, Massey University, Palmerston North 4442, New Zealand; m.c.kruger@massey.ac.nz; 3 Institute for Food, Nutrition and Well-being, University of Pretoria, Pretoria 0001, South Africa

**Keywords:** Peroxisome proliferator activated receptor, osteoclast, unsaturated fatty acid, RANKL signalling, osteoporosis

## Abstract

Osteoclasts are the sole bone resorbing cell in the body and their over activity is key in the development of osteoporosis. Osteoclastogenesis is mediated by receptor activator of nuclear factor κB ligand (RANKL) signalling pathways. Unsaturated fatty acids (UFA) are known to inhibit osteoclastogenesis by targeting RANKL signalling. However, the mechanisms of action remain unclear. Peroxisome proliferator activated receptors (PPARs) are a family of nuclear receptors, with three known isoforms (PPAR-α, PPAR-β/δ and PPAR-γ), that are known to bind UFAs and are expressed in osteoclasts. In this study, we aimed to determine how different families of UFAs activate PPARs and how PPAR activation influences osteoclast signalling. Human CD14+ monocytes were seeded into cluster plates with RANKL and macrophage colony stimulating factor (M-CSF) in the presence of PPAR agonists or different types of UFAs. All the PPAR agonists were shown to upregulate the activity of their respective receptors. Polyunsaturated fatty acids increased PPAR-α to a greater extent than monounsaturated fatty acids (MUFAs), which favoured PPAR-β/δ activation. All PPAR agonists inhibited osteoclastogenesis. The activation of RANKL signalling pathways and expression of key osteoclast genes were downregulated by PPAR agonists. This study reveals that PPAR activation can inhibit osteoclastogenesis through modulation of RANKL signalling.

## 1. Introduction

Osteoclasts are large multinucleated cells responsible for the resorption of bone [1]. Together with the bone forming osteoblasts, they maintain the health of bone by continually removing and replacing old bone in a process known as the bone remodelling cycle [2]. A breakdown in this cycle can lead to bone degenerative diseases such as osteoporosis, where osteoblasts cannot replace bone at the same rate at which osteoclasts resorb it [3]. Due to this higher rate of osteoclastic resorption in osteoporosis, the bone is often left brittle and prone to fracturing. Therefore, reducing osteoclast formation and activity may offer an approach to alleviating the symptoms of osteoporosis.

Osteoclasts are formed from precursors derived from the same haematopoietic stem cells that produce monocytes and macrophages [4]. These precursors will fuse and differentiate into mature osteoclasts in the presence of receptor activator of nuclear factor κB ligand (RANKL) and macrophage colony stimulating factor (M-CSF), which are produced by osteoblasts. M-CSF is responsible for cell survival and proliferation while RANKL plays a role in osteoclast differentiation and resorption [1]. RANKL binds to receptor activator of nuclear factor κB (RANK) on osteoclast precursors, which triggers a phosphorylation cascade within the cell. Nuclear factor κB (NF-κB) is activated through the phosphorylation of inhibitor of κB kinase (IKK), leading to the phosphorylation and degradation of Inhibitor of κB (IκB) [5]. This frees NF-κB to move into the nucleus and activate DNA binding sites. Simultaneously, the mitogen activated protein kinases (MAPKs), p-38, c-Jun N-terminal kinase (JNK) and extracellular signal–regulated kinase (ERK) are phosphorylated after RANK-RANKL binding [6]. MAPK and NF-κB signalling lead to the activation of nuclear factor of activated T-cells, cytoplasmic 1 (NFATc1), the master regulator of osteoclasts [7,8]. This will lead to the up-regulation of genes responsible for the formation and function of osteoclasts. RANKL signalling therefore offers up a potential target to inhibit osteoclast formation and activity and possibly treat bone degenerative diseases.

Peroxisome proliferator activated receptors (PPARs) are a family of nuclear receptors found in several tissues throughout the body. Three different isoforms of PPAR have been discovered, namely: PPAR-α, PPAR-β/δ and PPAR-γ. PPARs have been studied as therapeutic targets for several diseases. PPAR-γ agonists are currently in use for the treatment of type 2 diabetes [9] while PPAR-α agonists have been reported to favour an anti-atherogenic profile [10,11]. Dual agonists of PPAR-α and PPAR-γ have been developed to achieve insulin sensitising effects of PPAR-γ and high density lipoprotein cholesterol raising and triglyceride lowering effects of PPAR-α [12]. PPAR-α and PPAR-β/δ dual agonists have also been shown to lower triglycerides and low density lipoprotein cholesterol with concurrent insulin sensitising effects [13]. These results suggest that dual agonists may have potential in the treatment of metabolic syndrome, however, further clinical studies are necessary [14]. PPAR-α and PPAR-γ agonists have further shown β-amyloid reducing effects, which may offer potential in the treatment of Alzheimer’s disease [15,16]. Furthermore, several studies have evaluated the potential of PPAR-α and PPAR-γ as therapeutic targets for cancers [17]. Whereas PPAR-β/δ are associated with tumour progression [18], low levels of PPAR-α are associated with decreased survival in breast cancer [19]. PPAR-γ agonists show no anti-cancer effects when used alone, however, in combination with regulatory active anti-cancer drugs, PPAR-γ agonists may induce anakoinosis [20]. The therapeutic potential of PPARs in bone has proven controversial and needs further investigation.

All three PPAR isoforms are known to be expressed in osteoblasts and osteoclasts [21]. PPAR-γ has been shown to favour adipogenesis over osteogenesis in mesenchymal stem cells [22] and primary murine osteoblasts in vitro [23]. However, activation of either PPAR-α or PPAR-β/δ has been shown to promote osteoblastogenesis [21]. In osteoclasts, the activity of PPAR-γ has been controversial with some studies showing PPAR-γ promoting osteoclastogenesis [24], while other studies have shown it to decrease osteoclast formation [23,25]. PPAR-α and PPAR-β/δ have also shown interesting effects on osteoclast formation and activity. While both PPAR-α and PPAR-β/δ agonists inhibited osteoclast formation in differentiating cells, PPAR-α agonist failed to reduce resorption and PPAR-β/δ agonist actually increased resorption in mature osteoclasts [25]. Understanding how these receptors affect osteoclast signalling may help clarify these confounding results.

Unsaturated fatty acids (UFAs) are natural ligands of PPARs, and PPARs have been suggested as potential targets through which several UFAs exert their bone protective effects [26]. Both monounsaturated (MUFA) and polyunsaturated fatty acids (PUFA) are known to activate PPARs. The ω-7 MUFA palmitoleic acid (PLA) has been shown to induce PPAR-γ activity in human adipocytes to a greater extent than the PPAR-γ agonist, rosiglitazone [27]. Kliewer et al. reported that the ω-9 MUFA, oleic acid (OA) and the ω-6 PUFA, arachidonic acid (AA) can activate PPAR-α and PPAR-γ in CV-1 monkey fibroblasts [28]. The ω-3 PUFAs, docosahexaenoic acid (DHA) and eicosapentaenoic acid (EPA) have further been shown to activate PPAR-α activity in HeLa cervical carcinoma cells and rat primary hepatocytes [29,30]. However, which of the PPARs are activated by these different families of UFAs in osteoclasts is still unclear. Furthermore, how these PPARs influence osteoclast signalling remains unresolved. Therefore, this study sought to determine the effect of AA, EPA, DHA, PLA and OA on PPAR activation in a human osteoclast cell model. We further aimed to investigate the effects of PPAR activation on RANKL signalling in osteoclasts.

## 2. Results

### 2.1. PPARs are Expressed in CD14+ Monocytes

**Figure 1:**
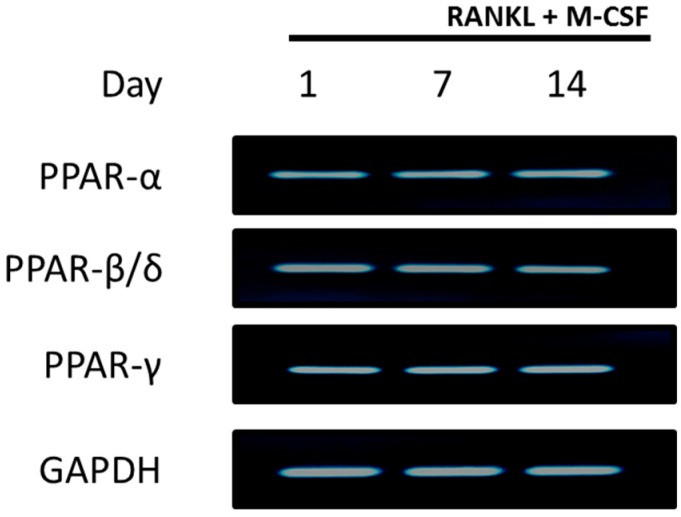
Peroxisome proliferator activated receptors (PPAR) expression in CD14+ monocytes. CD14+ monocytes were seeded into 24-well plates for 24 h to allow attachment. RNA was isolated for detection of PPAR expression at days 1, 7 and 14. GAPDH served as a loading control.

To test whether PPARs are expressed in differentiating CD14+ monocytes, cells were exposed to RANKL and M-CSF for 1, 7 or 14 days before RNA was extracted. PPAR expression was determined by PCR. Results indicate that all three PPARs, PPAR-α, PPAR-β/δ and PPAR-γ, were expressed in differentiating CD14+ monocytes from day 1 until day 14 of culture (Figure 1). Gene expression levels were shown to be similar among the three different PPARs at all the time points.

### 2.2. UFAs and PPAR Agonists do not Affect Cell Viability in Human CD14+ Monocytes

**Figure 2:**
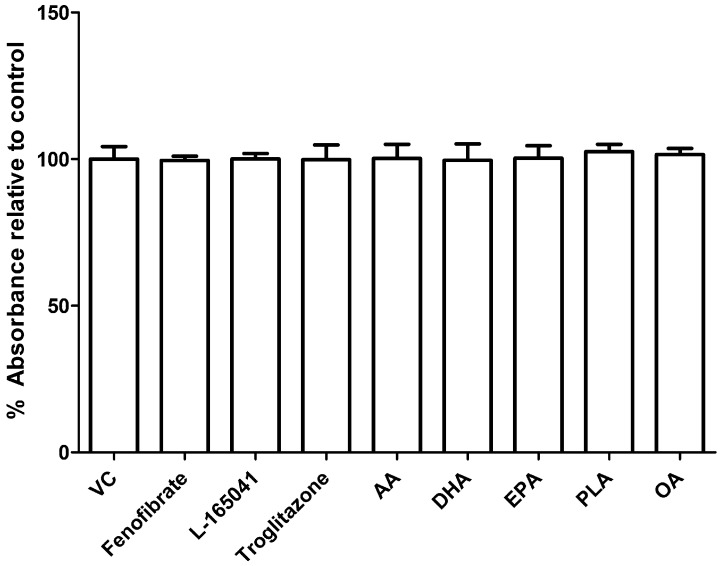
Effect of PPAR agonists and unsaturated fatty acids (UFAs) on cell viability in CD14+ monocytes. CD14+ monocytes were seeded into 96-well and exposed to PPAR agonists or UFAs. Cell viability was determined by resazurin assay. VC: vehicle control.

To test whether the PPAR agonists or UFA had any effects on cell viability in the undifferentiated human CD14+ monocytes, a resazurin assay was conducted. CD14+ monocytes were seeded into 96 well plates in the presence of fenofibrate (10 µM), L-165041 (10 µM), troglitazone (10 µM), AA (40 µM), DHA (40 µM), EPA (40 µM), PLA (100 µM) or OA (100 µM) for 48 h. Neither PPAR agonists nor any of the UFAs showed any effect on cell viability in the human CD14+ monocytes (Figure 2).

### 2.3. PPARs are Differentially Activated by Unsaturated Fatty Acids

**Figure 3:**
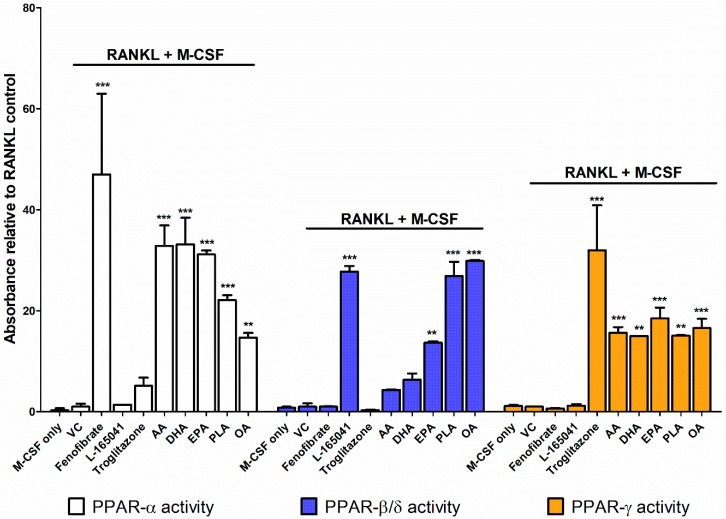
PPAR activity assay. CD14+ monocytes were seeded into 6-well plates and exposed to PPAR agonists or UFAs in the presence of receptor activator of nuclear factor κB ligand (RANKL) (30 ng mL^−1^) and macrophage colony stimulating factor (M-CSF) (25 ng mL^−1^). Nuclear protein was isolated and used to conduct a PPAR activity assay according to the manufacturer’s instructions. VC: vehicle control. ** *p* < 0.01, *** *p* < 0.001 vs. vehicle control.

CD14+ monocytes were seeded into 6-well plates in complete alpha-MEM to attach overnight before cells were exposed to PPAR agonists (10 µM), AA (40 µM), DHA (40 µM), EPA (40 µM), PLA (100 µM) or OA (100 µM) in the presence of RANKL and M-CSF. After 24 hours, nuclear protein was extracted and used to determine PPAR activity. Fenofibrate (PPAR-α agonist) caused an increase in PPAR-α activity (Figure 3). L-165041 (PPAR-β/δ agonist) significantly increased PPAR-β/δ activity. Troglitazone (PPAR-γ agonist) significantly increased PPAR-γ activity but also caused a slight but non-significant increase in PPAR-α activity. All the UFAs increased the activity of all the PPARs. However, PPAR-α activity was the highest in the samples exposed to the PUFAs (AA, DHA and EPA). PPAR-β/δ activity was highest in the samples exposed to MUFAs (OA and PLA) (Figure 3).

### 2.4. PPAR Agonists Modulate Osteoclast Formation

**Figure 4:**
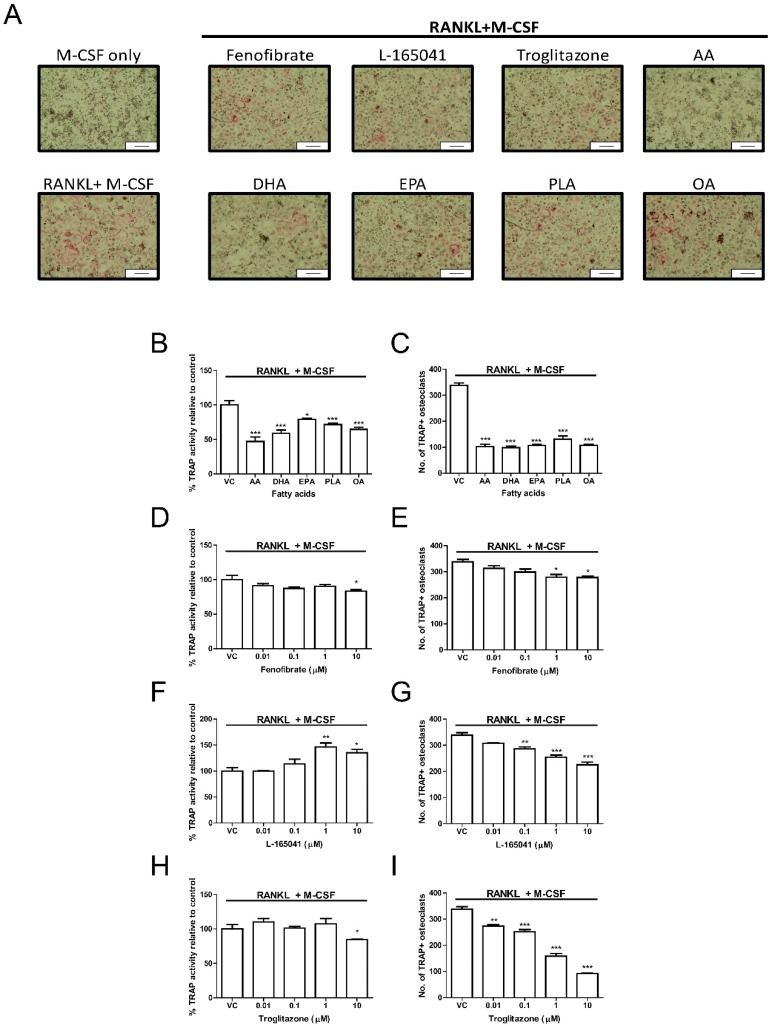
Effect of PPARs and UFAs on osteoclastogenesis. CD14+ monocytes were seeded into 96-well plates and exposed to PPAR agonists (10 µM) or UFAs in the presence of RANKL (30 ng mL^−1^) and M-CSF (25ng mL^−1^). (**A**) Cells were stained for tartrate resistant acid phosphatase (TRAP) and then visualized under a light microscope. TRAP positive cells appear pink. Scale bar = 0.2 mm. (**B,D,F,H**) TRAP activity was measured in the conditioned media using p-NPP as a substrate. (**C,E,G,I**) Quantification of TRAP positive osteoclasts. TRAP positive osteoclasts with three or more nuclei were counted. VC: vehicle control. * *p* < 0.05, ** *p* < 0.01, *** *p* < 0.001 vs. vehicle control.

Human CD14+ monocytes were seeded into 96-well plates in complete alpha-MEM and exposed to PPAR agonists or UFAs in the presence of RANKL and M-CSF for 14 days. Tartrate resistant acid phosphatase (TRAP) activity was determined from conditioned media using pNPP as a substrate. The cells were further fixed and stained for TRAP (Figure 4A). AA, DHA, EPA, PLA and OA significantly reduced TRAP activity (Figure 4B) and this corresponded with a decrease in osteoclast numbers (Figure 4C). Fenofibrate only significantly reduced TRAP activity at 10 µM (Figure 4D) but reduced osteoclast numbers at 1–10 µM (Figure 4E). Exposure to L-165041 resulted in contradictory findings. While L-165041 exposure significantly increased TRAP activity at 1–10 µM (Figure 4F), osteoclast numbers were significantly reduced at 0.1–10 µM (Figure 4G). Troglitazone resulted in a decrease in TRAP activity at 10 µM (Figure 4H). However, osteoclast numbers were reduced by troglitazone at all the concentrations tested (Figure 4I). For downstream experiments only 10 µM concentration was used for the PPAR agonists as this concentration decreased osteoclast numbers for all the PPAR agonists. 

### 2.5. PPAR Agonists Modulate RANKL Signalling in CD14+ Monocytes

**Figure 5:**
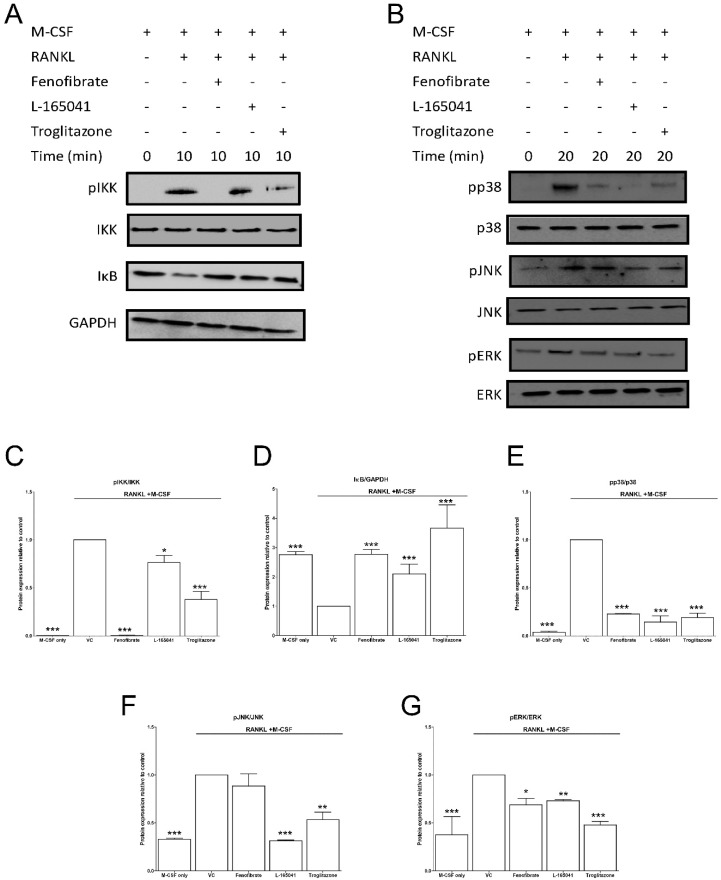
Effect of PPAR agonists on RANKL signalling. CD14+ monocytes were seeded into 6-well and exposed to PPAR agonists and M-CSF (25 ng mL^−1^) for 4 h before the addition of RANKL (30 ng mL^−1^) for 10–20 min. (**A**) The activation of NF-κB pathway proteins was determined by western blot. (**B**) Similarly, the activation of mitogen activated protein kinases (MAPK) proteins was determined. (**C**–**G**) Band densities were quantified using Image J software. VC: vehicle control. * *p* < 0.05, ** *p* < 0.01, *** *p* < 0.001 vs. vehicle control.

After confirming the UFAs could activate PPARs in our osteoclast model, we sought to determine whether activation of PPARs could modulate RANKL signalling pathways. CD14+ monocytes were seeded into 6-well plates in complete alpha-MEM to attach overnight before cells were exposed to PPAR agonists in the presence of M-CSF. RANKL was then added for 10–20 min. Protein was isolated and used to determine activation of proteins in the RANKL signalling pathway using western blotting. The addition of RANKL led to the phosphorylation of IKK and the degradation of IκB after 10 min (Figure 5A). All three agonists inhibited IKK phosphorylation and IκB degradation. Band densities were quantified, and these changes were determined to be statistically significant (Figure 5C,D). 

The activation of MAPK proteins (p38, JNK and ERK) was also determined (Figure 5B). All three agonists again resulted in a statistically significant inhibition of the phosphorylation of p38 and ERK (Figure 5E,G). Only L-165041 and troglitazone appeared to decrease JNK phosphorylation (Figure 5F).

### 2.6. PPAR Agonists Modulate Osteoclast Specific Gene Expression

**Figure 6:**
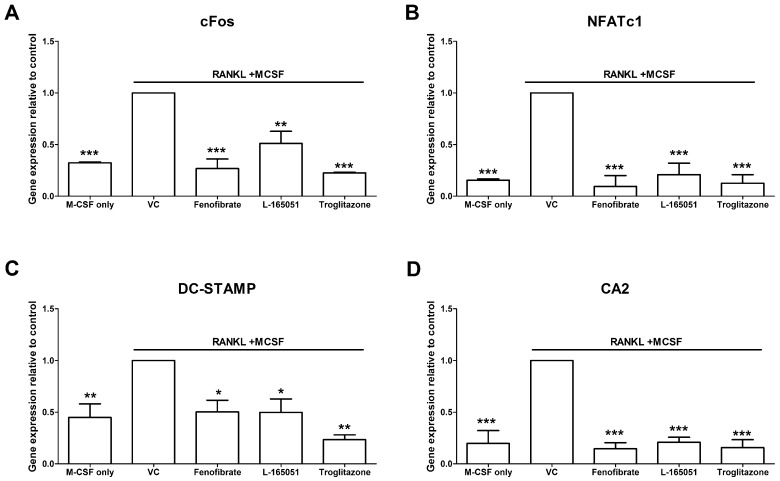
Effect of PPAR agonists on osteoclast specific gene expression. CD14+ monocytes were seeded into 24-well and exposed to PPAR agonists in the presence of RANKL (30 ng mL^−1^) and M-CSF (25 ng mL^−1^) for 14 days. Expression of (**A**) cFos, (**B**) NFATc1, (**C**) DC-STAMP and (**D**) CA2 was determined by qPCR. VC: vehicle control. * *p* < 0.05, ** *p* < 0.01, *** *p* < 0.001 vs. vehicle control.

CD14+ monocytes were seeded in complete alpha-MEM and exposed to PPAR agonists in the presence of RANKL (30 ng mL^−1^) and M-CSF (25 ng mL^−1^) for 14 days. Thereafter RNA was isolated, and osteoclast specific gene expression was determined by qPCR. The absence of RANKL resulted in significantly lower levels of cFos (Figure 6A), NFATc1 (Figure 6B), DC-STAMP (Figure 6C) and CA2 (Figure 6D) compared to the RANKL positive vehicle control. All three PPAR agonists were also shown to lower cFos, NFATc1, DC-STAMP and CA2 expression.

## 3. Discussion

There are three known isoforms of PPAR, namely: PPAR-α, PPAR-β/δ and PPAR-γ [31]. In this study, the activation of PPARs by UFAs and the effect of PPAR activation on RANKL signalling were investigated in a human primary osteoclast cell model. PPAR activity was evaluated as well as osteoclast formation and the activity of several signalling pathways. The purpose of this study was to understand how UFAs affect PPAR signalling and if activation of PPARs can affect osteoclast signalling. 

To determine the effects of PPARs on osteoclast signalling, human CD14+ monocytes were used as a primary osteoclast cell line. These cells are derived from the same stem cells as osteoclast precursors and are known to express RANK and c-fms, the receptors for RANKL and M-CSF respectively. They have been shown to be able to differentiate into osteoclasts in the presence of RANKL and M-CSF [32]. Furthermore, human peripheral blood mononuclear cells have been shown to express all three PPARs [25]. We confirmed in this study that the CD14+ monocytes express high levels of the PPARs from day 1 until day 14 of culture (Figure 1). The selectivity of PPAR agonists for their receptors was determined. We showed that fenofibrate, the PPAR-α agonist, significantly increased PPAR-α activity and did not affect PPAR-β/δ or PPAR-γ activity (Figure 3). L-165041, the PPAR-β/δ agonist, was shown to significantly increase PPAR-β/δ activity without affecting PPAR-α or PPAR-γ activity. Troglitazone, the PPAR-γ agonist, significantly increased PPAR-γ activity and, also to a lesser extent, PPAR-α activity. Other studies have noted that troglitazone can stimulate PPAR-α activity [33]. However, in our study troglitazone did not significantly increase PPAR-α activity and was therefore deemed acceptable to use. 

The different classes of UFAs showed varying effects on PPAR activity. The PUFAs, AA, DHA and EPA, showed greater increases in PPAR-α and PPAR-γ activity than PPAR-β/δ. Kliewer et al. have shown that AA and OA can activate PPAR-α and PPAR-γ in CV-1 monkey fibroblasts [28]. This study showed that AA and ALA induced higher levels of PPAR-α and PPAR-γ activation than OA [28]. DHA and EPA have further been shown to activate PPAR-α activity in HeLa cervical carcinoma cells and rat primary hepatocytes [29,30]. In the HeLa cells, DHA and EPA induced PPAR-α activity to a greater extent than OA and AA [29]. Interestingly, we showed that the MUFAs, PLA and EPA had higher PPAR-β/δ activity than the PUFAs. This may indicate that PPAR-β/δ has a stronger affinity for MUFAs in osteoclasts. Alternatively, this may be due to the varying concentrations used for the PUFAs and MUFAs in this present study. OA has been shown to induce gene expression of fatty acid handling genes in rat INS-1E pancreatic cells through PPAR-β/δ activation [34]. However, the INS-1E cells had significantly higher levels of PPAR-β/δ than PPAR-α and did not express PPAR-γ. Therefore, it is possible that OA may have activated PPAR-β/δ due to the decreased expression of PPAR-α and PPAR-γ. This present study made use of human CD14+ cells that were shown to express all three PPARs and the first to compare the activation of PPARs by UFAs in a human primary osteoclast cell line. PPAR-α activity was highest in cells exposed to the PUFAs while PPAR-β/δ activity was highest in cells exposed to the MUFAs. PPAR-γ activity was similar across all the different UFAs. However, the effect of these PPAR receptors on osteoclast signalling pathways was still unclear. Therefore, we further sought to determine how individual stimulation of these PPARs would influence the RANKL signalling pathways.

All three PPAR agonists significantly reduced osteoclast formation at 10 µM (Figure 4). Troglitazone, the PPAR-γ agonist, was shown to have the most potent anti-osteoclastogenic effect while fenofibrate, the PPAR-α agonist, was the least potent. Chan et al. did not observe a reduction in osteoclast formation by fenofibrate at 10 µM as seen in our present study [25]. This may be because Chan et al. made use of human peripheral blood mononuclear cells, which contain a mixture of different cell types whereas we made use of purified CD14+ monocytes. However, certain PPAR-α (GW9578), PPAR-β/δ (L-165041) and PPAR-γ (ciglitazone) agonists have been shown to decrease osteoclastogenesis in human peripheral blood mononuclear cells [25]. Interestingly, mature osteoclasts exposed to L-165041 were shown to increase resorption [25]. We report that the activity of TRAP, an enzyme highly expressed in mature osteoclasts [35], was increased by L-165041 in differentiating CD14+ monocytes (Figure 4F). TRAP is believed to be involved in the generation of reactive oxygen species needed for resorption and an increase in TRAP could be the cause of the increased resorption reported by Chan et al. Fenofibrate and troglitazone however decreased TRAP activity. Taken together, these results may indicate that PPAR-β/δ activation can have stimulatory effects on mature osteoclasts while having inhibitory effects on differentiating osteoclasts. 

Zou et al. reported that PPAR-γ knockdown did not affect osteoclast differentiation in vivo or in vitro [36]. Interestingly, bone marrow macrophages treated with rosiglitazone, a PPAR-γ agonist, showed an increase in the expression of cathepsin K, a key resorption enzyme [36]. Wan et al. have further shown that PPAR-γ activation may in fact promote osteoclast differentiation as PPAR-γ deficient mice were shown to develop osteopetrosis [24]. This may suggest that pharmacological activation of PPAR-γ may increase osteoclast formation but PPAR-γ may have no physiological effects on osteoclasts. Similar to Okazaki et al. [37], this present study showed that the PPAR-γ agonist, troglitazone, has anti-osteoclastogenic effects. Rosiglitazone has been shown to bind to PPAR-γ with a greater affinity than troglitazone [38], which may explain the differences between this study and that of Zou et al. Cho et al. have shown that rosiglitazone can inhibit osteoclast formation and resorption in murine bone marrow macrophages [23]. However, the study conducted by Zou et al. did not evaluate resorption in vitro and Cho et al. did not measure cathepsin K expression. Therefore, it is difficult to say whether these two studies present truly conflicting results. Furthermore, the decrease of resorption seen in the study by Cho et al. may be a result of decreased osteoclast formation and not a result of decreased expression of resorption genes. Nevertheless, these results indicate that much is still unclear about the effects of activation of these receptors in osteoclasts.

After RANKL binds to RANK, it leads to the phosphorylation of IKK, which in turn phosphorylates IκB, marking it for degradation. This frees NF-κB to cross the nuclear membrane and bind to DNA and trigger osteoclast differentiation [6]. Cho et al. showed that rosiglitazone prevented IκB degradation and inhibited NF-κB activation in bone marrow macrophages [23]. We have similarly shown that IκB degradation was inhibited by troglitazone (Figure 5). Moreover, troglitazone prevented the phosphorylation of IKK. Fenofibrate and L-165041 were also shown to decrease IKK phosphorylation and IκB degradation. RANKL-RANK interaction can also lead to the phosphorylation and activation of the MAPKs, p38, JNK and ERK [6]. This further amplifies the activation of osteoclast specific genes. We further showed that the phosphorylation of p38, JNK and ERK was inhibited by activation of any of the three PPARs (Figure 5). These results indicate, for the first time, that PPAR activation can modulate RANKL signalling pathways in a human osteoclast cell line. Furthermore, we evaluated the expression of downstream osteoclast specific genes (cFos, NFATc1, DC-STAMP and CA2). In this present study we report for the first time that the expression of cFos, NFATc1, DC-STAMP and CA2 can be inhibited by the activation of all three PPARs (Figure 6). Wan et al. have shown that PPAR-γ activation can inhibit the expression of key osteoclast regulating genes such as cFos, NFATc1 and CA2 [24]. NFATc1 is the master regulator of osteoclasts and is upregulated by cFos [7]. Activation of NFATc1 will lead to the fusion of pre-osteoclasts, which is mediated by DC-STAMP [35]. Furthermore, NFATc1 stimulates the production of enzymes involved in resorption such as MMP-9, CTSK and CA2 [35]. 

Previous studies have shown that PPAR-γ agonists can inhibit osteoclast formation but favour adipogenesis at the expense of osteoblastogenesis [39]. Furthermore, several PPAR-γ agonists, such as rosiglitazone, ciglitazone, pioglitazone, and troglitazone have been discontinued in clinical practice due to reported increases in the risk of liver disease [40]. Therefore, even though they may have anti-osteoclastogenic effects, the use of PPAR-γ agonists may decrease bone formation and have an overall negative effect on health. PPAR-α agonists have been shown to inhibit osteoblast formation in favour of adipogenesis but to a lesser extent than PPAR-γ agonists [39]. However, other studies have shown that PPAR-α agonists can promote osteoblast differentiation [41] and protect against ovariectomized induced bone loss [42]. These confounding results may be due to differences in experimental setup and underlie the need for more research into the effects of these agonists in bone. PPAR-β/δ agonists do not induce adipogenesis [43]. Future studies should focus on PPAR-α and PPAR-β/δ agonists, which may be more promising targets for protecting bone health than PPAR-γ. As the UFAs were shown to activate PPARs in our cell model, it may be speculated that the UFAs can modulate osteoclast formation and function through these PPARs. Interestingly, PPARs were able to inhibit activation of cytoplasmic proteins. PPARs are nuclear receptors and this may indicate the use of co-activators and second messengers to elicit their effects on osteoclasts. Further studies are needed to elucidate the signalling mechanisms used by the PPARs in osteoclasts.

## 4. Materials and Methods

### 4.1. Reagents and Materials

DHA, AA, EPA, OA, PPAR agonists (PPAR-α: fenofibrate; PPAR-β/δ: L-165041; PPAR-γ: troglitazone), antibiotic solution (100 μg mL^−1^ streptomycin, 0.25 μg mL^−1^ fungizone and 100 µg mL^−1^ penicillin), and all other chemicals were supplied by Sigma-Aldrich (St Louis, MO, USA). PLA was provided by Santa Cruz Biotech (Dallas, TX, USA). DNAse and RNAse free alpha-MEM was provided by GIBCO (Grand Island, NY, USA). Foetal bovine serum (FBS) was supplied by Amersham (Little Chalfont, UK). Human RANKL and M-CSF were purchased from R&D Systems (Minneapolis, MN, USA). LASEC (Cape Town, South Africa) supplied all cell culture plates and other plasticware. Sigma-Aldrich (St Louis, MO, USA) or Abcam (Cambridge, UK) supplied the primary antibodies used in this study. All primers were synthesised by InqabaBiotec (Pretoria, South Africa).

### 4.2. Preparation of Fatty Acids and Agonists

Stock concentrations (100 mM) of AA, DHA, EPA, PLA and OA were prepared in ethanol. PPAR agonists were prepared in DMSO at a stock concentration of 10 mM. The compounds were frozen at −70 °C in aliquots and diluted to working concentrations in culture media when required. AA, DHA and EPA were used at concentrations that have previously been shown to inhibit osteoclast formation (40 µM) [44]. Similarly, PLA and OA were used at concentrations that have been shown to have anti-osteoclastogenic effects (100 µM) [45,46]. PPAR agonists were used at 0.01–10 µM concentration [25]. DMSO in the media did not exceed 0.1% and this was used as the vehicle control. 

### 4.3. Ethics Statement

This study received ethical clearance from the Faculty of Health Sciences Research Ethics Committee, University of Pretoria (reference number: 321/2017) (approval date: 31/08/2017).

### 4.4. CD14+ Monocyte Isolation

Peripheral blood was drawn from healthy male donors (age 18–30) after informed consent as previously reported [44]. In brief: Blood was diluted 1:1 in PBS supplemented with 2 mM EDTA. Diluted blood was then carefully layered on a Histopaque® gradient and centrifuged at 450 xg for 30 min at 20 °C without brake in a Hettich Rotixa 120R centrifuge (Kirchlengern, Germany). Peripheral blood mononuclear cells (PBMCs) were then carefully collected from the layer between the plasma and the Histopaque® layers. Cells were washed with PBS supplemented with 2 mM EDTA and centrifuged at 300 xg for 10 min at 20 °C twice. After a cell count, CD14+ monocytes were sorted by magnetic separation. The cells were resuspended in 80 µl of PBS supplemented with 2 mM EDTA and 0.5% BSA per 10^7^ cells and 20 µl of MACS® MicroBeads (Miltenyi Biotec, Bergisch Gladbach, Germany) per 10^7^ cells were added. The suspension was mixed carefully and incubated for 15 min on ice with mixing by gentle inversion every 5 min. Thereafter the cells were washed with PBS supplemented with 2 mM EDTA and 0.5% BSA followed by centrifugation at 300 xg for 10 min. The cells were resuspended in PBS supplemented with 2 mM EDTA and 0.5% BSA and passed through a magnetic separation column. Thereafter, 1 mL of the PBS supplemented with 2 mM EDTA and 0.5% BSA was added and the magnetically labelled cells were flushed out of the column. The CD14+ monocytes were then counted and seeded into cell cluster plates at 1.3 × 10^5^ cells cm^−2^ in complete alpha-MEM (alpha-MEM containing 10% heat-inactivated FBS and 1% antibiotic solution).

### 4.5. PPAR Expression

PCR was conducted to determine the expression of PPAR-α, PPAR-β/δ, and PPAR-γ in the CD14+ monocytes during different stages of maturation. CD14+ monocytes were seeded into 24-well plates at 1.3 × 10^5^ cells cm^−2^ in complete alpha-MEM. After 24 hours, 7 days and 14 days, total RNA was extracted using TRI Reagent® (Sigma-Aldrich, St Louis, MO, USA) and reverse transcribed into cDNA using M-MuLV reverse transcriptase as previously described [44]. PCR was conducted using KAPA2G Robust HotStart Ready Mix (KAPA biosystems. Wilmington, MA, USA) and a PxE 0.2 Thermal Cycler (Thermo Fischer Scientific, Waltham, MA, USA) using the following cycling protocol: initial denaturation at 95 °C for 3 min, denaturation at 95 °C for 10 s, annealing at 60 °C for 15 s, and extension at 72 °C for 15 s for 35 cycles and final extension at 72 °C for 1 min. The products were resolved on a 1% agarose in TAE buffer gel at 150 V and visualized with ethidium bromide using a gel documentation system attached to a monochrome scientific grade camera (E- Box 1000/26M, Vilber Lourmat, Collégien, France). The primers used are shown in Table 1.

### 4.6. Resazurin Assay

A resazurin assay was conducted to determine whether the UFAs or PPAR agonists had cytotoxic effects on human CD14+ monocytes. Resazurin is a blue dye that is converted to resorufin, a pink dye, by metabolically active cells. This colourimetric change can be used as a measure of cell viability. Cells were seeded at 1.3 × 10^5^ cells cm^−2^ in 96-well plates in complete alpha-MEM for 24 h before exposure exposed to AA (40 µM), DHA (40 µM), EPA (40 µM), PLA (100 µM), OA (100 µM), fenofibrate (10 μM), L-165041 (10 μM) or troglitazone (10 μM) for 48 h. At the end of the culture period, media was discarded from each well and replaced with 10 µl of 0.8 μM resazurin solution in 90 μl of fresh media. The plates were then incubated at 37 °C for 4 h and absorbance was read at 570 nm using 600 nm as a reference using an Epoch micro-plate spectrophotometer (BioTek, Winooski, VT, USA).

### 4.7. PPAR Activation Assay

#### 4.7.1. Nuclear Fractionation

CD14+ monocytes were seeded at a density of 1.3 × 10^5^ cells cm^−2^ in 6-well plates and incubated at 37 °C overnight. Cells were then exposed to PPAR agonists or fatty acids and RANKL (30 ng mL^−1^) and M-CSF (25 ng mL^−1^) for 24 h. At the end of culture, the cells were washed in ice-cold PBS before lysis with cytoplasmic extraction buffer (10 mM HEPES, 60 mM KCl, 1 mM EDTA, 0.0075% (v/v) NP40, 1 mM DTT and 1 mM PMSF. pH adjusted to 7.6). Cells were then incubated on ice for 3 min followed by centrifugation at 150 xg at 4 °C for 4 min. The supernatant was removed and gently resuspended in cytoplasmic extraction buffer without NP40. Following centrifugation at 150 xg at 4 °C for 4 min, the supernatant was removed, and the pellet was resuspended in nuclear extraction buffer (20 mM Tris, 420 mM NaCl, 1.5 mM MgCl2, 0.2 mM EDTA, 1 mM PMSF. pH adjusted to 8). After 10 min incubation on ice, the extract was centrifuged at maximum speed at 4 °C for 10 min. The supernatant (containing the nuclear fraction) was then transferred to a fresh tube. 

#### 4.7.2. PPAR Assay

PPAR activation was determined using a PPAR (alpha, delta, gamma) Transcription Factor Assay Kit (Abcam, Cambridge, UK) according to the manufacturer’s instructions. In brief, nuclear protein extracts were loaded into wells coated with PPAR-α, PPAR-β/δ or PPAR-γ consensus DNA and the plates were stored at 4 °C overnight. The plates were then washed 5 times with wash buffer to remove unbound reagents. Primary antibody was added, and the plates were incubated at room temperature for 60 min. After another wash period, goat anti-rabbit-HRP conjugate secondary antibody was added and the plates were stored at room temperature for an hour. Thereafter, transcription factor developing solution was added for 15–45 min followed by the addition of stop solution. The plates were then read at 450 nm using an Epoch micro-plate spectrophotometer (BioTek, Winooski, VT, USA).

### 4.8. Differentiation of CD14+ Monocytes

CD14+ monocytes were seeded in 96-well plates at 1.3 × 10^5^ cells cm^−2^ in complete alpha-MEM in the presence of RANKL (30 ng mL^−1^), M-CSF (25 ng mL^−1^) and PPAR agonists (0.01–10 µM) or AA (40 µM), DHA (40 µM), EPA (40 µM), PLA (100 µM) or OA (100 µM). Medium and all factors were replaced every 2–3 days. Experiments were terminated on day 14.

#### 4.8.1. TRAP Activity

TRAP activity in the media was measured as previously described [44]. In brief, conditioned medium was incubated for an hour in TRAP solution (6 mM pNPP, 25 mM disodium tartrate. pH adjusted to 5.5) at 37 °C. Stop solution (0.3 M NaOH) was added and absorbance was read at 405/650 nm used using an Epoch micro-plate Spectrophotometer (BioTek, Winooski, VT, USA).

#### 4.8.2. TRAP Stain

After fixation with 3.7% formaldehyde in PBS, cells were stained for TRAP as previously described [47]. Cells were incubated in 0.1 M sodium tartrate in 0.2 M acetate (pH adjusted to 5.2) for 5 min at 37 °C followed by incubation with 20 mg mL^−1^ naphthol AS-BI phosphate in the acetate-tartrate solution at 37 °C for 30 min. The solution was replaced with hexazotised pararosaniline in acetate-tartrate solution for 15 min at 37 °C before counter-staining with haematoxylin for 40 sec. Osteoclasts appear as large multinucleated cells staining red. TRAP-positive stained cells with three or more nuclei per unit area were counted as mature osteoclasts [48]. Photomicrographs were taken with an Olympus SC30 camera attached to an Olympus BH2 microscope (Olympus, Tokyo, Japan). 

### 4.9. Western Blot

CD14+ monocytes were seeded at a density of 1.3 × 10^5^ cells cm^−2^ in 6-well plates in complete alpha-MEM supplemented with M-CSF (25 ng mL^−1^) and incubated at 37 °C overnight. Cells were exposed to PPAR agonists for 4 h. RANKL (30 ng mL^−1^) was then added and cells were incubated for 15 min.

Cells were then washed in ice-cold PBS before being lysed in 100 µl RIPA buffer (150 mM NaCl, 5 mM EDTA, 50 mM Tris, 1% Triton X-100, 0.5% sodium deoxycholate, 0.1% SDS in dH_2_O, pH adjusted to 8) supplemented with 0.3 M PMSF, 5% protease inhibitor cocktail and 5% phosphatase inhibitor cocktail. The lysate was centrifuged at 13 000 xg for 30 min using a Jouan Br4i centrifuge (DJB Labcare, Buckinghamshire, UK) at 4 °C for the removal of non-lysed fragments. Purified proteins were quantified using a BCA protein determination kit (Pierce Biotechnology, Rockford, IL, USA) according to the manufacturer’s instructions. Equal concentrations of protein were loaded in sample buffer containing 1% β-mercaptoethanol and resolved on a 12% polyacrylamide gel. Bio-Rad transfer system (Bio-Rad, Hercules, CA, USA) was used to transfer proteins onto a nitrocellulose membrane with Tris-glycine transfer buffer (192 mM glycine, 25 mM Tris, and 20% methanol). After blocking with 5% BSA powder for an hour the membranes were washed with TBS-T and then incubated with rabbit polyclonal primary antibodies against IκB, pIKK, IKK, JNK, pJNK, ERK, pERK, p38 and pp38 (1:1 000) overnight at 4 °C. After incubation with goat anti-rabbit IgG Antibody, HRP-conjugate (1:20 000) (Sigma-Aldrich, St Louis, MO, USA) for an hour at room temperature, the membranes were developed using a Clarity ECL Western Blotting Substrate (Biorad, Hercules, CA, USA) and visualized on a ChemiDoc MP (Bio-Rad, Hercules, CA, USA). ImageJ software were used to quantify band densities [49].

### 4.10. Quantitative PCR

To determine whether PPAR agonists influenced the expression of osteoclast specific genes, a qPCR was conducted. CD14+ monocytes were seeded at a density of 1.3 × 10^5^ cells cm^−2^ in 24-well plates in complete alpha-MEM in the presence of RANKL (30 ng mL^−1^) and M-CSF (25 ng mL^−1^) and PPAR agonists (0.01–10 µM). Medium and all factors were replaced every 2–3 days. Experiments were terminated on day 14.

RNA was collected and reverse transcribed as described previously. For quantitative PCR (qPCR) the SensiFAST™ SYBR® No-ROX kit Master Mix (Bioline Reagents, London, UK) was used for amplification using the following cycling protocol: initial denaturation at 95 °C for 2 min, denaturation at 95 °C for 5 s, annealing at 65 °C for 10 s, and extension at 72 °C for 10 s for 40 cycles. A LightCycler® Nano System (Roche Diagnostics, Basel, Switzerland) was used for detection. Relative gene expression levels were analysed using the 2^−ΔΔCT^ method and results were normalized to the housekeeping gene (GAPDH). The primers used are shown in Table 2.

### 4.11. Statistical Analysis

Data displayed is representative of three repeats unless otherwise stated. Results are displayed as a mean ± the standard deviation relative to the vehicle control. Data was compared to the vehicle control of the respective experiment and analysed by a one-way analysis of variance (ANOVA) followed by a Bonferroni post hoc test using GraphPad software (La Jolla, CA, USA). All p-values ≤ 0.05 were considered statistically significant.

## Figures and Tables

**Table 1:** Primers used for PPAR expression.

Gene	Forward Primer Sequence (5′–3′)	Reverse Primer Sequence (5′–3′)
GAPDH	CATGTTGCAACCGGGAAGG	CGCCCAATACGACCAAATCA
PPAR-α	TCATCAAGAAGACGGAGTCG	CGGTTACCTACAGCTCAGAC
PPAR-β/δ	GCCCTTTGTGATCCACGACA	GGATGCTCTTGGCGAACTCAG
PPAR-γ	ATGACAGCGACTTGGCAATA	GCAACTGGAAGAAGGGAAAT

**Table 2:** Primers used for qPCR.

Gene	Forward Primer Sequence (5′–3′)	Reverse Primer Sequence (5′–3′)
GAPDH	CATGTTGCAACCGGGAAGG	CGCCCAATACGACCAAATCA
cFos	CCCATCGCAGACCAGAGC	ATCTTGCAGGCAGGTCGGT
NFATc1	GTGGAGAAGCAGAGCAC	ACGCTGGTACTGGCTTC
DC-STAMP	ATGACTTGCAACCTAAGGGCAAAG	GTCTGGTTCCAAGAAACAAGGTCAT
CA2	GAGTTTGATGACTCTCAGGACAA	CATATTTGGTGTTCCAGTGAACCA

## Abbreviations

AA	Arachidonic acid
CA2	Carbonic anhydrase 2
DC-STAMP	Dendritic cell-specific transmembrane protein
DHA	Docosahexaenoic acid
EPA	Eicosapentaenoic acid
ERK	Extracellular signal–regulated kinase
IκB	Inhibitor of κB
IKK	Inhibitor of κB kinase
JNK	c-Jun N-terminal kinase
MAPK	Mitogen activated protein kinases
M-CSF	Macrophage colony stimulating factor
MUFA	Monounsaturated fatty acid
NF-κB	Nuclear factor κB
NFATc1	Nuclear factor of activated T-cells, cytoplasmic 1
OA	Oleic acid
PBMC	Peripheral blood mononuclear cells
PLA	Palmitoleic acid
PPAR	Peroxisome proliferator activated receptor
PUFA	Polyunsaturated fatty acid
RANK	Receptor activator of nuclear factor κB
RANKL	Receptor activator of nuclear factor κB ligand
TRAP	Tartrate resistant acid phosphatase
UFA	Unsaturated fatty acid
